# Pharmacokinetic Genes Do Not Influence Response or Tolerance to Citalopram in the STAR*D Sample

**DOI:** 10.1371/journal.pone.0001872

**Published:** 2008-04-02

**Authors:** Eric J. Peters, Susan L. Slager, Jeffrey B. Kraft, Greg D. Jenkins, Megan S. Reinalda, Patrick J. McGrath, Steven P. Hamilton

**Affiliations:** 1 Department of Psychiatry and Institute for Human Genetics, University of California San Francisco, San Francisco, California, United States of America; 2 Department of Health Sciences Research, Mayo Clinic, Rochester, Minnesota, United States of America; 3 Department of Psychiatry, Columbia University College of Physicians and Surgeons and New York State Psychiatric Institute, New York, New York, United States of America; James Cook University, Australia

## Abstract

**Background:**

We sought to determine whether clinical response or tolerance to the Selective Serotonin Reuptake Inhibitor (SSRI) citalopram is associated with genetic polymorphisms in potentially relevant pharmacokinetic enzymes.

**Methodology:**

We used a two-stage case-control study design in which we split the sample of 1,953 subjects from the Sequenced Treatment Alternatives to Relieve Depression (STAR*D) trial into a discovery (n = 831) and validation set (n = 1,046). Fifteen polymorphisms from five (CYP2D6, ABCB1, CYP2C19, CYP3A4, and CYP3A5) pharmacokinetic genes were genotyped. We examined the associations between these polymorphisms and citalopram response and tolerance. Significant associations were validated in the second stage for those polymorphism found to be statistically significant in the first stage.

**Conclusions:**

No genetic polymorphism in the pharmacokinetic genes examined was significantly associated with our response or tolerance phenotypes in both stages. For managing pharmacological treatment with citalopram, routine screening of the common pharmacokinetic DNA variants that we examined appears to be of limited clinical utility.

## Introduction

Significant inter-individual variation exists in clinical response to and tolerance of antidepressant medication. Common genetic variation may be partly responsible for these phenotypic differences. The use of genotype information in clinical psychopharmacology could potentially help clinicians avoid the standard trial and error approach, and allow a more efficient way to maximize efficacy and minimize toxicity [1]–[3] as is done in certain situations with cancer treatment [4].

Drug metabolism and transport genes such as CYP2D6 and CYP2C19 are obvious pharmacogenetic candidate genes given their known interaction with drugs like selective serotonin reuptake inhibitors (SSRI) and their metabolites *in vivo* [5]. Moreover, several of these pharmacokinetic genes harbor common variants that have been shown to impair enzyme function [6]. For example, Yin et al. found that homozygous carriers of the non-functional allele of CYP2C19 show a 42% decrease in clearance of the SSRI citalopram compared to that of homozygous carriers of the wild type allele [7]. Gräsmader et al. showed that plasma concentrations of several antidepressants were significantly influenced by CYP2D6 and CYP2C19 genotype, however, clinical response was not associated with plasma concentrations of these drugs [8].

Despite these known *in vivo* relationship between antidepressant medications and pharmacogenetic genes, few epidemiological studies investigating the relationship between antidepressant response and pharmacokinetic gene variants have been carried out. In a naturalistic, retrospective study of 28 patients who experienced adverse events and 16 patients that were non-responsive to a variety of antidepressants, the authors observed an association with CYP2D6 genotype [9]. In a prospective study of 246 elderly subjects taking the SSRI paroxetine, CYP2D6 genotype was not associated with side effect burden [10]. Another recent study of 100 depressed subjects taking fluvoxamine showed that CYP2D6 genotype does not influence the frequency of gastrointestinal side effects, although when CYP2D6 genotype is combined with a serotonin 2A receptor polymorphism, the authors did observe such an association [11]. Despite the equivocal results of these studies, some investigators have advocated the use of pharmacokinetic enzyme variant information to guide clinical therapy of SSRI, particularly by adjustment of the dose prescribed [12], [13]. Even though there is intuitive appeal in ascribing differences in drug tolerance and efficacy to variation in pharmacokinetic genes [13], [14] no adequately powered studies have been published that consistently report a significant clinical effect. Here, we investigate the potential role of five pharmacokinetic genes on the response to and tolerance of citalopram using a large clinical sample of depressed patients who were enrolled in the Sequenced Treatment Alternatives to Relieve Depression (STAR*D) study [15].

## Materials and Methods

### Sample

Subjects are those who enrolled in STAR*D and consented to give DNA (N = 1,953). The STAR*D trial was a large NIMH-sponsored treatment trial involving 4,041 subjects that was designed to assess effectiveness of antidepressant treatments in generalizable samples, and to determine outcomes for outpatients with non-psychotic major depressive disorder (MDD) treated with citalopram. The study design and methods for this clinical trial are reviewed in [16], and further demographic information on the cohort that consented to give DNA has been previously published [17]. The aim of STAR*D was to prospectively determine which of a number of treatments are beneficial for subjects experiencing an unsatisfactory response to citalopram. To increase the generalizability of the findings, STAR*D utilized broad inclusion criteria and enrolled an ethnically diverse population [15]. Diagnosis was made using the Psychiatric Diagnostic Screening Questionnaire, and depressive symptoms were assessed with the 16-item Quick Inventory of Depressive Symptomatology (Self-Report [QIDS-SR] version) [18] collected at baseline and at all clinic visits. Subjects meeting inclusion criteria and providing consent were administered citalopram for a target trial of 12 weeks of treatment with vigorous dosing (20–60 mg/day). The subset of subjects who provided DNA samples was 61.8% female and was 78.1% Caucasian, 16.1% African-American, and 5.8% other races [19]. Hispanics accounted for 14.0% of the sample. The average citalopram dose at study exit was 45.5 mg (S.D. = 15.7). Subjects were consented for genetic studies as part of the National Institute of Mental Health's Human Genetic Initiative and the work described here was approved by the institutional review board of the University of California, San Francisco.

### Phenotypic definitions

We defined six phenotypes to evaluate citalopram response and tolerability. The first two were **responders** and **non-responders**: responders are subjects who had at least 42 days of treatment and whose QIDS-SR score on their final clinical visit shows ≥50% reduction in score compared to baseline; the remaining subjects, who also had at least 42 days of treatment, were then considered non-responders. The ≥50% reduction in symptom severity on the HRSD_17_ is the conventional definition of response in clinical trials. We used the QIDS-SR score to estimate severity since all subjects had this rating and it correlates highly with the 17-item Hamilton Rating Scale for Depression (HRSD_17_) score [18]. We required this 42 day threshold to ensure an adequate exposure to citalopram and to enhance the power to find associations between genotype and response by reducing potential heterogeneity. The third phenotype was **remission**, defined as a final QIDS-SR score ≤5. Our **specific** response phenotype is based on our attempt to further reduce heterogeneity by attempting to separate placebo response from true drug response in antidepressant trials [20]. Some response to antidepressant medication is a placebo response, which we posit may have either no genetic determinant or a different genetic substrate than “true” drug response. Thus it is of interest to limit our definition of response to true pharmacologic response rather than placebo response. For these phenotypes, a “specific” pattern of response was defined by persistence, or the maintenance of response for the remainder of the study once it was attained. Previous studies considered “specific” patterns to be further characterized by delayed response, i.e., after the first two weeks [21]. We were unable to employ this criterion because the STAR*D study design did not include ratings before week two of treatment, except for baseline scores. We defined persistent, or “**specific**” responders, as those subjects who had a sustained response at all consecutive visits following the first visit with response, as measured by ≥50% reduction in QIDS-SR scores. Those whose response occurred only at the last visit were removed from the analysis. Note that “specific” responders are a subset of responders (as defined by the response phenotype above). Moreover, because visits were at least two weeks apart, we assumed that intervening weeks were characterized by the response defined by the previous visit. Our tolerance outcome was based on study exit data; all patients who continued with citalopram at the end of STAR*D Level 1 treatment were considered **tolerant**, while patients who refused to continue citalopram or left the study at any time due to side effects were considered **intolerant**. For those who left Level 1 for further treatment but did not want to continue with citalopram, their phenotype was probably tolerant, probably intolerant, or intolerant based on the level of side effects at the study exit based on the Global Rating of Side Effect Burden [22]. In order to reduce heterogeneity, we did not use subjects who were considered probably tolerant or probably intolerant. The mean duration of treatment was 11.10 weeks for non-responders, 12.42 weeks for responders, and 12.39 weeks for remitters. For the tolerance phenotype, the mean duration was 6.62 weeks for intolerant subjects and 12.14 weeks for tolerant subjects.

### Molecular methods

Several cytochrome P450 genes (CYP2C19, CYP2D6, CYP3A4, CYP3A5) are thought to be involved in the metabolism of citalopram [23]. We chose to examine DNA variants in cytochrome P450 genes that cause or are suspected to cause severe functional changes in the targeted proteins. However, for CYP3A enzyme activity, there are no known functional polymorphisms, thus we investigated two common SNPs in these genes. Indeed, CYP3A enzyme activity may play a small role in citalopram pharmacokinetics, as co-administration of the CYP3A inhibitor ritonavir does not substantially alter citalopram pharmacokinetics [24]. While citalopram is lipophilic and can cross the blood brain barrier without transport to some degree, the transporter P-glycoprotein (ABCB1) has been shown in animal models to contribute to the efflux of citalopram from the brain [25]. Therefore, we investigated three common SNPs in the ABCB1 gene (C1236T, G2677T, and C3435T) that have been associated with treatment outcome in acute myeloid leukemia patients and reduced P-glycoprotein expression *in vivo* [26], [27]. Due to the low population frequency of the more recently described G2677A allele (<2% in Caucasians), this variant was not genotyped in the present study [28].

Patients were genotyped for CYP3A5*3C, all three CYP2C19 variants (*2, *3, *17), and all three MDR1 variants using 5′ exonuclease fluorescence (Taqman) assays. CYP2D6*5 deletion status was determined using a previously published tetra-primer long range PCR assay [29]. All other CYP2D6 alleles (*3, *4, *6, *7, *8, *9) were determined by first specifically amplifying the CYP2D6 gene as a 5.1 kb long range PCR product (as described in [29]) followed by direct sequencing of two regions containing exons 3–4 and exons 5–6. This two step amplification procedure was preformed in order to avoid non-specific amplification of the CYP2D6 pseudogene located near the CYP2D6 gene. CYP3A4*1B genotype was determined by direct sequencing of a 320 bp PCR product that specifically amplifies the 5′ proximal region of the CYP3A4 gene [30]. PCR protocols and primer sequences are available upon request, and a synopsis of the 15 variants is shown in Table S1. Direct sequencing genotypes were scored using Mutation Surveyor v2.61. Genotype data has been deposited with the NIMH Center for Collaborative Genetic Studies on Mental Disorders (http://nimhgenetics.org/).

### Statistical methods

To reduce Type I error, we relied on a two-stage design for analysis [31]. Within each ethnic group, gender, and response to citalopram (using only our responders and nonresponders phenotypes), we randomly split our subjects *a priori* into a discovery set and validation set. Within each set, we stratified all analyses by self-reported ethnicity due to the large allele frequency differences and phenotype prevalence differences between ethnic groups. Only the two largest ethnic groups (Caucasian and African-American) were analyzed. Hardy-Weinberg equilibrium was evaluated for each SNP within the discovery set using all participants within each ethnic group. This is because all subjects had depression, and we do not suspect the variants to influence risk of depression. No SNPs were found to violate Hardy-Weinberg equilibrium using a Bonferroni-corrected threshold. We used unconditional logistic regression analysis to examine associations between each genetic polymorphisms and each phenotypic comparison. Comparisons performed were: 1) *responders* vs. *non-responders*, 2) *remitters* vs. *non-responders*, 3) *specific responders* vs. *non-responders*, and 4) *tolerant* vs. *intolerant*. Each polymorphism was modeled individually as gene-dosage effects in the regression models, and odds ratios (OR) and 95% confidence intervals were estimated. For the CYP2D6 and CYP2C19 genes, we also modeled the putative metabolism status of the subjects as follows. Individuals with two non-functional alleles in these genes were considered poor metabolizers (PMs), all other genotypes were considered extensive metabolizers (EM). Association between haplotypes and the phenotypes were calculated using a score test implemented in the computer program HAPLO.SCORE [32]. Pair-wise interactions among all independent SNPs were tested using logistic regression. A likelihood ratio test was used to test for significance of the interaction effect. Only those SNPs with a p-value of <0.05 from the single SNP analyses in the discovery set were evaluated in the validation set. Those SNPs in the validation set that had a p-value <0.05 and the same directionality of association as that in the screening set were reported as statistically significant. We used survival analysis to examine whether metabolizer status influenced the ability to complete the trial. Survival curves were generated by the method of Kaplan-Meier, and differences between PM and EM curves were tested using the log rank statistic. We also examined the relationship between metabolizer status and citalopram dose, comparing final dose between extensive and poor metabolizers at the CYP2D6 and CYP2C19 loci with a t-test. No correction for multiple comparisons was applied to our association tests. The two-stage design helps to control Type I error by requiring nominal statistical significance in both stages to be ultimately declared significant. The probability of a variant being significant at the 0.05 level in both stages is expected to be low.

## Results

Patients were genotyped for 15 polymorphisms in the CYP2D6, CYP2C19, CYP3A4, CYP3A5, and ABCB1 genes. We compared genotype frequencies between responders and non-responders, remitters and non-responders, and specific responders and non-responders within each racial subgroup. Note that remitters and specific responders are subsets of responders. We also compared genotype frequencies of subjects intolerant to citalopram to those who could tolerant the medication. Table 1 displays the frequency distribution of the phenotypes by ethnicity among subjects for the discovery and validation sets. Because one of our criteria for splitting our sample was based on the response/non-response phenotypes, the distribution of response and non-response are similar between the discovery and validation set. In the discovery set, we found seven variants to be associated (p<0.05) with citalopram response or tolerance. All but one of these were found in the African-American ethnic group. However, none of these SNPs were replicated in our validation set (Table 2). It is of note that the point estimates for the odds ratios for nearly all of these variants switched directionality in the second stage, most likely as a result of small samples sizes and the low allele frequencies of those variants. Similar non-significant results were obtained using haplotype testing (results not shown). CYP2D6 or CYP2C19 metabolizer status (PM vs. EM) was also not associated with citalopram response or tolerance in the first stage (results not shown). We also found no evidence for interaction (P>0.05) between the variants in any of the genes tested (results not shown). We further sought to determine if metabolizer genotype was correlated with other clinical variables of interest, namely the dosage of citalopram and the length of time a subject would continue with citalopram treatment. For all subjects, regardless of outcome or length of trial, dose was not correlated with CYP2D6 or CYP2C19 metabolizer status (see Table 3). Additionally, CYP2C19 or CYP2D6 metabolizer status did not significantly influence the subject's ability to remain in the trial (P = 0.65 and P = 0.95, respectively).

**Table 1:** Sample sizes of the discovery and validation subsets for each racial subgroup.

Variable	Discovery Set								Validation Set							
	Pheno 1 a		Pheno 2 b		Pheno 3 c		Pheno 4 d		Pheno 1 a		Pheno 2 b		Pheno 3 c		Pheno 4 d	
	Yes	No	Yes	No	Yes	No	Yes	No	Yes	No	Yes	No	Yes	No	Yes	No
	N(%)	N(%)	N(%)	N(%)	N(%)	N(%)	N(%)	N(%)	N(%)	N(%)	N(%)	N(%)	N(%)	N(%)	N(%)	N(%)
**Ethnicity**																
African-American	66	63	52	63	40	63	89	9	64	58	48	58	37	58	86	13
	(51.2)	(48.8)	(45.2)	(54.8)	(38.8)	(61.2)	(90.8)	(9.2)	(52.5)	(47.5)	(45.3)	(54.7)	(39.0)	(61.0)	(86.9)	(13.1)
Caucasian	395	257	331	257	287	257	514	51	404	254	348	254	272	254	554	125
	(60.6)	(39.4)	(56.3)	(43.7)	(52.8)	(47.2)	(91.0)	(9.0)	(61.4)	(38.6)	(57.8)	(42.2)	(51.7)	(48.3)	(81.6)	(18.4)

a, Responder vs. Non-Responder

b, Remitter vs. Non-Responder

c, Specific Responder vs. Non-Responder

d, Tolerant vs. Intolerant

**Table 2:** Single locus results for tests that were significant (p<0.05) in the discovery sample set.

Ethnicity	Phenotypic Comparison	Gene	Variant	Discovery set p-value (OR, 95% CI)	Validation set p-value (OR, 95% CI)
Caucasian	Tolerant vs. Intolerant	CYP2C19	*2	0.005 (0.44, 0.24–0.81)	0.86 (1.00, 0.63–1.57)
African-American	Responder vs. Non-Responder	ABCB1	C3435T	0.01 (0.36, 0.17–0.75)	0.59 (1.51, 0.70–3.26)
African-American	Remitter vs. Non-Responder	ABCB1	C3435T	0.02 (0.36, 0.16–0.78)	0.85 (1.28, 0.56–2.93)
African-American	Specific Responder vs Non-Responder	CYP2D6	*5	0.03 (4.44, 1.07–18.39)	0.32 (0.45, 0.09–2.37)
African-American	Specific Responder vs Non-Responder	CYP2D6	*4	0.04 (0.26, 0.05–1.23)	0.96 (1.24, 0.35–4.41)
African-American	Specific Responder vs Non-Responder	ABCB1	C3435T	0.02 (0.40, 0.17–0.93)	0.71 (1.39, 0.58–3.35)
African-American	Tolerant vs. Intolerant	CYP3A5	*3	0.04 (0.32, 0.08–1.37)	0.33 (1.57, 0.48–5.07)

Significance was assessed using logistic regression, and odds ratios (OR) and confidence intervals (CI) shown are for minor allele carrier versus non-carrier. No variants were significantly associated in both the discovery and validation sample sets.

**Table 3:** Effect of subject metabolizer status on final citalopram dose.

Metabolizer Status	Mean Final Dose (s.d.)	p-value
CYP2C19 EM	45.3 (15.7)	0.13
CYP2C19 PM	40.7 (16.4)	
CYP2D6 EM	45.4 (15.8)	0.25
CYP2D6 PM	43.2 (16.8)	

Mean final dose (mg) for each metabolizer group is shown, along with the standard deviation (S.D.) and significance level assessed using student's t-test. Results are shown for the Caucasian subgroup, similar non-significant results were obtain in the African-American subset.

## Discussion

There is growing interest in the utility of pharmacokinetic gene polymorphism screening in psychopharmacological treatment, particularly with antipsychotic medications and older antidepressant agents [6]. Others have argued that the efficacy and toxicity of most psychotropics could be influenced by DNA variants in pharmacokinetic genes, and that drug selection and dosage should ideally be based on genotypic information [12], [13]. There is growing consensus that there is little data that suggests that assessment of cytochrome P450 polymorphisms may be clinically useful for guiding SSRI therapy [33].

The flat dose-response curve and wide therapeutic index of SSRIs argue against a strong relationship between plasma levels and clinical response [34] and there is little evidence regarding how plasma levels of citalopram influence clinical efficacy [35]. This appears to be the case for citalopram, which has few drug-drug interactions based on *in vitro* and *in vivo* studies [23]. Nevertheless, the pharmacokinetics of many SSRIs, including citalopram, are affected by CYP2D6 and CYP2C19 genotype status, as polymorphisms in these enzymes do alter citalopram disposition [7], [36]–[38]. For example, CYP2C19 poor metabolizers showed a 42% decrease in citalopram clearance when compared to homozygous extensive metabolizers, yet there was no difference in the subject's side effect profile [7]. In another study of seven non-responders to citalopram, six of seven were extensive metabolizers for CYP2D6 and all seven were CYP2C19 extensive metabolizers [39]. When given an inhibitor of these two enzymes, citalopram serum levels rose in all seven subjects, with six of them showing substantial clinical improvement. These data suggest that enzymes involved in citalopram metabolism may contribute to response, at least in some extensive metabolizers. There are no similar data regarding side effects, although a large (n = 749) Swedish study found no difference in citalopram or desmethylcitalopram levels between those experiencing a number of common side effects compared and those who did not, suggesting that side effects are influenced primarily by pharmacodynamic rather than pharmacokinetic factors [40]. A study by Murphy et al. also found pharmacodynamic gene variation to be important in antidepressant intolerance [10]. Recently, a study was reported involving genotyping of ABCB1 variants in persons taking antidepressants in which an association between several of these variants and response in ∼114 persons taking ABCB1 substrates, but not in ∼85 persons taking drugs that are not substrates for the protein encoded by ABCB1 [41]. The three ABCB1 variants that we genotyped for this report were genotyped by Uhr et al., and just as in our study, no association with response phenotypes were noted. For the eleven SNPs found to be associated by Uhr et al., ten are in very strong linkage disequilibrium. Three of these markers were genotyped as part of our unpublished genome-wide association study of antidepressant response in our sample, and these three adequately tag the ten correlated markers based on CEU HapMap Phase II data (r^2^≥0.8 or = 1.0 for seven or five of seven remaining markers, respectively). These three markers, rs10280101, rs2235040, and rs12720067, showed p-values of 0.58, 0.30, and 0.56, respectively, for the remission phenotype. Urh et al. reported one additional marker as being associated with antidepressant response. This marker, rs2235015, was not genotyped by us, nor did we genotype a SNP tagging this marker. Thus it is an open question if this last marker shows association to treatment response phenotypes in our sample.

The size of the STAR*D study provides a clinical sample with statistical power to detect moderately sized genetic influences. In this study, we detected no significant association between any of the polymorphisms and our treatment phenotypes. Our two-stage analysis allowed us to control Type I error by requiring validation of our results in a second sample. However, by splitting our sample as such, we sacrificed statistical power, and thus increased the risk of Type II error. For our response phenotype in the discovery set, we had 80% power to detect a minimum detectable odds ratio of 1.9 assuming an allele frequency of 0.05 and 5% significance level using our Caucasian sample. The minimum detectable odds ratio increased to 2.74 for the tolerance phenotype. There are multitudinous potential analyses that can be carried out given the richness of the phenotypic data. In this study, we did not formally correct our results for multiple comparisons, although our two-stage design serves to control Type I error, lending further support to the overall negative results. The availability of our genotype data at the NIMH Center for Collaborative Genetic Studies on Mental Disorders facilitates additional exploratory hypothesis testing.

Our study has several limitations. Given the many differences in SNP allele frequencies and phenotype classification among self-reported ethnic groups, population stratification may be a potential explanation for our negative findings, with true associations being obscured by unobserved population sub-structure. This is particularly relevant given the wide differences in allele frequencies between populations for many of the genes studied here [42]. However, population studies have found that self-reported ethnicity is a close surrogate for underlying genetic ancestry information [43], thus we sub-grouped our analyses based on self-reported ethnicity in order to limit potential confounding. By analyzing the ethnicity groups separately and using a two-stage association approach, we had reduced power to detect associations in the African American subgroup, and thus cannot entirely dismiss these loci in this subgroup. We limited our genotyping of pharmacokinetic candidate genes to known, deleterious alleles that are common in Caucasian populations. In order to comprehensively screen these genes, rare and functionally unknown variants would need to be genotyped. The STAR*D clinical study, while large and broad in scope, was not explicitly designed for pharmacogenetic studies of this type. For instance, citalopram was chosen partly due to its lower potential for influence by pharmacokinetic polymorphism. Citalopram dosage was also not fixed, though the majority of subjects (78%) were receiving 40–60 mg per day at the end of the study. The final citalopram dosage prescribed was not influenced by the subject's genotype status. This is consistent with work carried out with many of the same functional DNA variants in the Clinical Antipsychotic Trials of Intervention Effectiveness (CATIE) study, in which there was no association to dosing, efficacy, or tolerability to five antipsychotics (David Goldstein, personal communication). This observation is particularly interesting in that others have noted a strong correlation between maximum prescribed dose of phenytoin or carbemazepine in epilepsy and genetic variants in CYP2C9 or SCN1A , suggesting clinical adjustment of dose in response to genotype [44]. Reflecting the “real-world” treatment focus of the STAR*D study, patients were not drug naïve and certain concomitant medications for general medical conditions were allowed during treatment. Unfortunately, systematic data on concomitant medications was not collected during the trial, and thus we were unable to control for this theoretical drug-drug interaction effect. It is noteworthy that the analysis of the CATIE study indicates that using concomitant medications known to alter metabolic status did not alter the results (David Goldstein, personal communication). While clinical outcome, not alteration of pharmacokintetic profiles, was our study endpoint, circulating concentrations of citalopram or citalopram metabolites would have been a useful proxy measure of compliance. Unfortunately, plasma citalopram levels were not obtained from any STAR*D subjects, thus unmeasured compliance is a limitation of this study and consequently reduces our statistical power. The design of genetics component of STAR*D was not entirely prospective, with some subjects consenting for DNA collection any time after initiating treatment, raising the possibility that those consenting for genetic analysis do not represent all subjects. We adjusted our analyses for the time period between starting the trial and donating blood for DNA, and found no effect on the results (data not shown). Finally, our findings regarding citalopram may not be generalizable to other SSRI's, each of which has a unique metabolic disposition. Any broadly administered pharmacogenetic test will have to tolerate similar limitations in order to be useful in “real-world” clinical settings. Thus, at least for citalopram, it may be premature to advocate pharmacokinetic gene analysis for dose adjustment or clinical decision making.

## Supporting Information

Table S1 (0.06 MB DOC)
