# Supplementary material for: Pharmacokinetic Genes Do Not Influence Response or Tolerance to Citalopram in the STAR*D Sample
Source: PLoS One. 2008 Apr 2;3(4):e1872. doi: 10.1371/journal.pone.0001872 (PMC2268970; doi:10.1371/journal.pone.0001872)
Supplement: Table S1 — (0.06 MB DOC) [file pone.0001872.s001.doc]

Table S1.

List of genotyped variants in pharmacokinetic genes. Variants are listed by gene, accepted nomenclature (see http://www.cypalleles.ki.se/), minor allele frequencies in the STAR*D sample, and known functional status of the variant. PM, poor metabolizer, as described in Materials and Methods. Note that opposite alleles are minor alleles for ABCB1 C3435T (rs1045642) between Caucasians (C) and African-American (T) samples.

| **Gene** | **Variant** | **Caucasian** | **African-American** | **Enzyme function in vivo** |
| --- | --- | --- | --- | --- |
| CYP2D6 | *3 | 0.02 | 0.003 | none |
| CYP2D6 | *4 | 0.19 | 0.07 | none |
| CYP2D6 | *5 | 0.03 | 0.06 | none |
| CYP2D6 | *6 | 0.01 | 0.003 | none |
| CYP2D6 | *7 | 0.0003 | 0.002 | none |
| CYP2D6 | *8 | - | - | none |
| CYP2D6 | *9 | 0.03 | 0.005 | decreased |
| CYP3A4 | *1B | 0.04 | 0.65 | unknown |
| CYP3A5 | *3C | 0.09 | 0.70 | decreased |
| CYP2C19 | *2 | 0.13 | 0.19 | none |
| CYP2C19 | *3 | - | 0.02 | none |
| CYP2C19 | *17 | 0.21 | 0.20 | increased |
| ABCB1 | C1236T (rs1128503) | 0.44 | 0.19 | unknown |
| ABCB1 | G2677T (rs2032582) | 0.44 | 0.08 | unknown |
| ABCB1 | C3435T (rs1045642) | 0.51 | 0.21 | unknown |
|  |  |  |  |  |
| CYP2D6 | PM | 0.05 | 0.02 | none |
| CYP2C19 | PM | 0.02 | 0.02 | none |
